# Comparison of the Diagnostic Performance of C26:0-Lysophosphatidylcholine and Very Long-Chain Fatty Acids Analysis for Peroxisomal Disorders

**DOI:** 10.3389/fcell.2020.00690

**Published:** 2020-07-29

**Authors:** Yorrick R. J. Jaspers, Sacha Ferdinandusse, Inge M. E. Dijkstra, Rinse Willem Barendsen, Henk van Lenthe, Wim Kulik, Marc Engelen, Susan M. I. Goorden, Frédéric M. Vaz, Stephan Kemp

**Affiliations:** ^1^Department of Clinical Chemistry, Laboratory Genetic Metabolic Diseases, Amsterdam UMC, Amsterdam Gastroenterology and Metabolism, University of Amsterdam, Amsterdam, Netherlands; ^2^Department of Pediatric Neurology, Amsterdam UMC, Amsterdam Leukodystrophy Center, Emma Children’s Hospital, Amsterdam Neuroscience, University of Amsterdam, Amsterdam, Netherlands

**Keywords:** adrenoleukodystrophy, peroxisomes, dried bloodspots, C26:0-lysophosphatidylcholine, very long-chain fatty acids, VLCFA, beta-oxidation

## Abstract

Peroxisomes are subcellular organelles that are involved in various important physiological processes such as the oxidation of fatty acids and the biosynthesis of bile acids and plasmalogens. The gold standard in the diagnostic work-up for patients with peroxisomal disorders is the analysis of very long-chain fatty acid (VLCFA) levels in plasma. Alternatively, C26:0-lysophosphatidylcholine (C26:0-LPC) can be measured in dried blood spots (DBS) using liquid chromatography tandem mass spectrometry (LC-MS/MS); a fast and easy method but not yet widely used. Currently, little is known about the correlation of C26:0-LPC in DBS and C26:0-LPC in plasma, and how C26:0-LPC analysis compares to VLCFA analysis in diagnostic performance. We investigated the correlation between C26:0-LPC levels measured in DBS and plasma prepared from the same blood sample. For this analysis we included 43 controls and 38 adrenoleukodystrophy (ALD) (21 males and 17 females) and 33 Zellweger spectrum disorder (ZSD) patients. In combined control and patient samples there was a strong positive correlation between DBS C26:0-LPC and plasma C26:0-LPC, with a Spearman’s rank correlation coefficient of *r* (114) = 0.962, *p* < 0.001. These data show that both plasma and DBS are suitable to determine blood C26:0-LPC levels and that there is a strong correlation between C26:0-LPC levels in both matrices. Following this, we investigated how VLCFA and C26:0-LPC analysis compare in diagnostic performance for 67 controls, 26 ALD males, 19 ALD females, and 35 ZSD patients. For C26:0-LPC, all ALD and ZSD samples had C26:0-LPC levels above the upper limit of the reference range. For C26:0, one out of 67 controls had C26:0 levels above the upper reference range. For 1 out of 26 (1/26) ALD males, 1/19 ALD females and 3/35 ZSD patients, the C26:0 concentration was within the reference range. The C26:0/C22:0 ratio was within the reference range for 0/26 ALD males, 1/19 ALD females and 2/35 ZSD patients. Overall, these data demonstrate that C26:0-LPC analysis has a superior diagnostic performance compared to VLCFA analysis (C26:0 and C26:0/C22:0 ratio) in all patient groups. Based on our results we recommend implementation of C26:0-LPC analysis in DBS and/or plasma in the diagnostic work-up for peroxisomal disorders.

## Introduction

Peroxisomes are organelles that are involved in various important physiological processes such as the oxidation of fatty acids and the biosynthesis of bile acids and plasmalogens (Wanders et al., 2010). Peroxisomal disorders affect 1 in 5.000 individuals (Waterham et al., 2016). These disorders can be divided into two subgroups: peroxisome biogenesis disorders and disorders caused by a single peroxisomal enzyme deficiency (Klouwer et al., 2016). Peroxisome biogenesis disorders result from a faulty assembly of peroxisomes and include Zellweger spectrum disorders (ZSD), peroxisomal fission defects, and rhizomelic chondrodysplasia punctata (RCDP) type 1 and 5.

Zellweger spectrum disorder are autosomal recessive disorders caused by mutations in one of 13 *PEX* genes. ZSD are characterized by a large variety in clinical presentation (Klouwer et al., 2015). Symptoms can include neurological dysfunctions, adrenal insufficiency, and hearing and vision problems. Peroxisomal single enzyme deficiencies include among others, acyl-CoA oxidase deficiency (Ferdinandusse et al., 2007), *ACBD5* deficiency (Ferdinandusse et al., 2017), and the most common peroxisomal disorder, adrenoleukodystrophy (ALD) (Moser et al., 2001). Many of these disorders are characterized by the accumulation of very long-chain fatty acids (VLCFAs; ≥C22:0). ALD is the result of a defect in the *ABCD1* gene (Mosser et al., 1993) and is characterized by a highly unpredictable clinical manifestation (Kemp et al., 2016). The *ABCD1* gene encodes for a peroxisomal membrane protein, referred to as ALDP, that is involved in the transport of VLCFAs into the peroxisome, where they are broken down via β-oxidation (Singh et al., 1984). A non-functional ALDP results in accumulation of VLCFAs in body fluids and tissues (Moser et al., 1981).

Analysis of VLCFA levels is the gold standard in the diagnostic work-up for peroxisomal disorders. The most commonly used methods are based on gas chromatography (GC), which includes the method developed by Moser et al. (1981) and its improved version in 1991 (Moser and Moser, 1991). Alternatively, methods based on stable isotope dilution using GC combined with mass spectrometry (GCMS) are also widely used (Vreken et al., 1998). While these methods are highly reproducible, they are time-consuming and labor intensive. Sample preparation can take up to 2 days while analysis takes up to 30 min per sample using GCMS. Although VLCFAs are regarded as the most important biomarkers for most peroxisomal disorders, false negative results have been reported in approximately 15–20% of women with ALD (Moser et al., 1999). These factors have led to the development of an alternative diagnostic test: the analysis of C26:0-lysophosphatidylcholine (C26:0-LPC) in dried bloodspots (DBS) using liquid chromatography tandem mass spectrometry (LC-MS/MS) or flow injection analysis mass spectrometry (FIA-MS) (Hubbard et al., 2006; Turgeon et al., 2015; Haynes and Jesús, 2016). C26:0-LPC is elevated in bloodspots from patients with impaired VLCFA metabolism, including women with ALD with normal plasma VLCFA levels (Huffnagel et al., 2017). Importantly, its analysis is considerably faster when compared to VLCFA analysis (Hubbard et al., 2009; Huffnagel et al., 2017; Klouwer et al., 2017). In fact, the identification of C26:0-LPC as a specific and sensitive biomarker in bloodspots was of paramount importance for the initiation of ALD newborn screening (Turgeon et al., 2015; Moser et al., 2016; Barendsen et al., 2020).

In the current diagnostic landscape C26:0-LPC in DBS is used for ALD newborn screening. However, the gold standard in the diagnostic work-up for patients with peroxisomal disorders is the analysis of plasma VLCFA levels. The objectives of our study were: (1) To investigate the correlation of C26:0-LPC in DBS and C26:0-LPC in plasma, and (2) To compare the diagnostic performance of C26:0-LPC analysis and VLCFA analysis. We discuss the important factors in the consideration of these methods and provide an overview of possible discrepancies.

## Materials and Methods

### Patient Samples

All routine measurements of C26:0-LPC and VLCFA performed at the Laboratory Genetic Metabolic Diseases in the Amsterdam UMC between June 2018 and June 2019 were collected and used for this study. This set was expanded with measurements in additional ALD and ZSD samples. This resulted in samples from 67 controls, 26 ALD males, 19 ALD females and 35 ZSD patients. All ALD patients had confirmed *ABCD1* mutations, 32 ZSD patients had confirmed *PEX1* mutations, 2 ZSD patients had confirmed *PEX6* mutations and 1 ZSD patient had a confirmed *PEX26* mutation. Control samples consisted of samples that were screened for a peroxisomal disorder and resulted in a negative outcome. All samples were collected according to the institutional guidelines for blood sampling.

### Sample Preparation C26:0-LPC

Analysis of C26:0-LPC was performed as described earlier by Van de Beek et al. (2016). Briefly, for DBS, a single punch of a 6.2 mm (1/4 inch) bloodspot was extracted with 10 μL of an internal standard solution containing 1 μmol/L D_4_-C26:0-lysoPC in 0.5 mL of methanol by ultrasonication for 5 min in a sonicator bath (Branson 3510) at room temperature. For plasma, 10 μL was extracted with 10 μL of an internal standard solution containing 1 μmol/L D_4_-C26:0-lysoPC in 0.5 mL of acetonitrile by ultrasonication for 5 min in a sonicator bath (Branson 3510) at room temperature. After centrifugation (5 min, 14000 RPM) the resulting methanol (DBS) or acetonitrile (plasma) layer was transferred to a new glass tube and evaporated under a constant stream of nitrogen at 40°C. The samples were then reconstituted in 50 μL methanol, transferred to a sample vial, and capped.

### HPLC-MS/MS Analysis

Samples were injected using an ACQUITY UPLC system (Waters, Milford, MA, United States) on a 50 × 2.1 mm, 2.6 μm particle diameter Kinetex C8 column (Phenomenex, Torrance, CA, United States). The column was held at a constant temperature of 50°C. The composition of mobile phase A was 0.1% formic acid in water and mobile phase B was 0.1% formic acid in methanol. The gradient used was as follows: *T* = 0 min: 36% A, 64% B, flow 0.4 mL/min toward *T* = 6 min: 0% A, 100% B, flow 0.4 mL/min; *T* = 6–11 min: 0% A, 100% B, flow 0.4 mL/min, and *T* = 11–11.1 back to 36% A, 64% B, flow 0.4 mL/min. Detection was done using a Quattro Premier XE (Waters, Milford, MA, United States) using electrospray ionization in positive mode. The source temperature was 130°C, and capillary voltage was 3.5 kV. Multiple reaction monitoring (MRM) was done on masses 636.50 > 104.10 and 640.50 > 104.10 with a dwell time of 0.03 s. Argon was used as a collision gas.

### Sample Preparation VLCFAs

Very long-chain fatty acids analysis was performed essentially as described earlier by Vreken et al. In a glass tube, 100 μL plasma was added to 100 μL internal standard solution dissolved in toluene that consisted of 50 μmol/L D_4_-C22:0, 50 μmol/L D_4_-C24:0, 1 μmol/L D_4_-C26:0. Acidic hydrolysis was performed by adding 2 mL of a 0.5 mol/L HCl in acetonitrile and incubating at 110°C for 45 min. After cooling to room temperature, fatty acids were extracted into 4 mL of hexane. The resulting hexane layer was transferred to a new glass tube and washed with 3.5 mL 1M KOH. Next, the hexane layer was removed and 600 μL 25% HCl was added. Fatty acids were extracted with 4 mL of hexane. The hexane layer was then transferred to a new glass tube and dried under nitrogen at 50°C. Fatty acids were derivatized with 50 μL pyridine and 50 μL MTBSTFA at 80°C for 30 min. The solvent was then evaporated under nitrogen at 50°C and the derivatized fatty acids were reconstituted in 200 μL hexane and transferred to autosampler vials.

### GCMS Analysis

Analysis was performed on an Agilent 7890B GC using a 5977A MSD mass spectrometer for compound detection. Separation was achieved on a Cp-sil 19, 25m^∗^0.25mm^∗^0.20 μm column. Temperature gradient was as follows: 60°C was held for 1.5 min after which the temperature increased to 240°C at a rate of 10°C/min. The temperature was then increased to 270, at 4°C/min. Finally the temperature increased to 300°C at 20°C/min. An injection volume of 1 μL was used in splitless mode using a splitless time of 1.5 min. The carrier gas was Helium (50 kPa). Single ion monitoring mode was used for detection of VLCFAs.

## Results

### C26:0-LPC in DBS Versus Plasma

**FIGURE 1:**
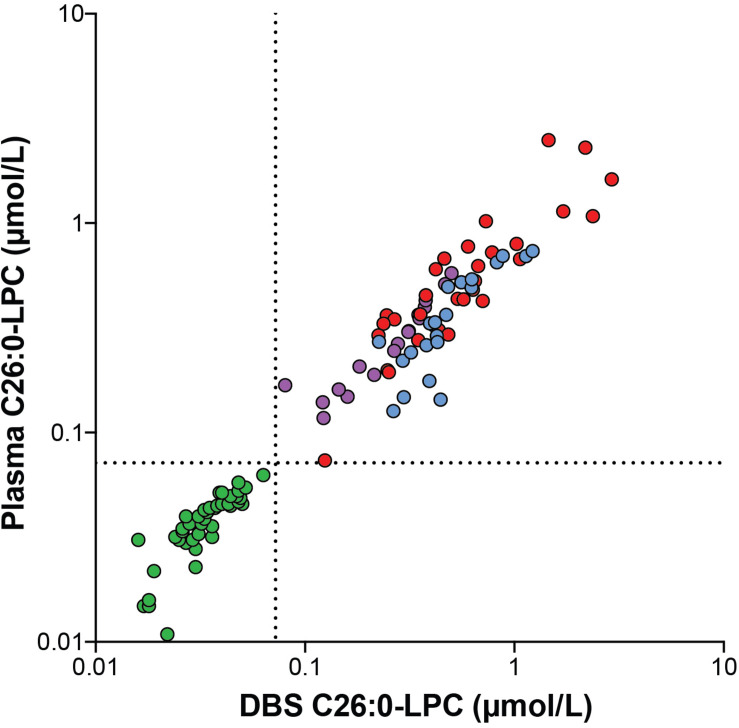
Correlation plot showing the correlation between C26:0-LPC levels analyzed in DBS and plasma samples from controls (green circles, *n* = 43), ALD males (blue circles, *n* = 21), ALD females (purple circles, *n* = 17) and ZSD patients (red circles, *n* = 33) that were generated from the same blood sample. The upper limit of the reference range (0.072 μmol/L) is indicated by the dashed lines.

To investigate the correlation between C26:0-LPC in DBS and C26:0-LPC in plasma we measured C26:0-LPC in DBS and plasma prepared from the same blood sample. For this analysis we included 43 control, 21 ALD males, 17 ALD females and 33 ZSD samples (Figure 1). The median C26:0-LPC level in DBS of controls was 0.033 μmol/L (range 0.016–0.063 μmol/L), in ALD males it was 0.425 μmol/L (range 0.224–1.208 μmol/L), in ALD females it was 0.276 μmol/L (range 0.080–0.497 μmol/L) and in ZSD patients it was 0.470 μmol/L (range 0.124–2.881 μmol/L). The upper limit of the reference range in our laboratory is 0.072 μmol/L. All patients had elevated levels of C26:0-LPC. There was a clear separation between controls and patients; the highest control level was 0.063 μmol/L and the lowest patient value was 0.080 μmol/L in a sample from an ALD female. For C26:0-LPC in plasma, the median level in controls was 0.040 μmol/L (range 0.011–0.063 μmol/L), in ALD males it was 0.333 μmol/L (range 0.127–0.736 μmol/L), in ALD females it was 0.266 μmol/L (range 0.118–0.576 μmol/L) and in ZSD it was 0.445 μmol/L (range 0.074–2.485 μmol/L). All patients had elevated levels of C26:0-LPC. The highest control level was 0.063 μmol/L and the lowest patient level was 0.074 μmol/L in a sample from a ZSD patient. We investigated the correlation between C26:0-LPC levels measured in DBS and C26:0-LPC levels measured in plasma with a Spearman’s rank-order correlation. In the combined set of both control and patient samples there was a very strong positive correlation between DBS C26:0-LPC and plasma C26:0-LPC with a Spearman’s rank correlation coefficient of *r* (114) = 0.962, *p* < 0.001. For controls alone, it was *r* (43) = 0.907, *p* < 0.001. For patients alone, it was *r* (71) = 0.861, *p* < 0.001. These data show that both plasma and DBS are suitable to determine blood C26:0-LPC levels and that there is a strong correlation between C26:0-LPC levels in both matrices. The strong correlation between plasma and DBS allowed for combining plasma and DBS C26:0-LPC data for comparison to VLCFAs as a diagnostic marker for peroxisomal disorders.

### C26:0-LPC Levels vs. VLCFA

**FIGURE 2:**
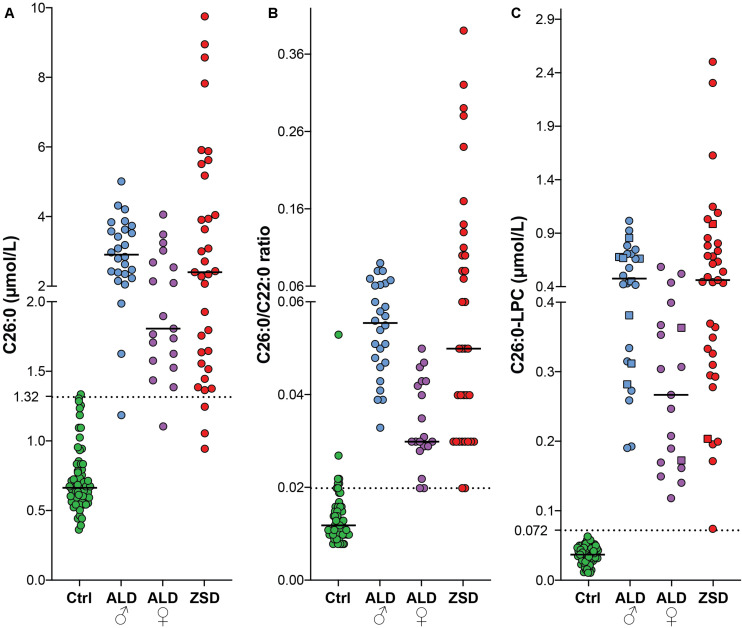
Scatterplots of C26:0 (plasma) **(A)**, C26:0/C22:0 ratio (plasma) **(B)** and C26:0-LPC [DBS (squares) and plasma (circles)] **(C)** from controls (green, *n* = 67), ALD males (blue, *n* = 26), ALD females (purple, *n* = 19) and ZSD patients (red, *n* = 35). The upper limit of the reference range for C26:0 (1.32 μmol/L), the C26:0/C22:0 ratio (0.02) and C26:0-LPC in DBS (0.072 μmol/L) is indicated by the dashed lines.

To compare the diagnostic performance of plasma VLCFA analysis (C26:0 concentration and C26:0/C22:0 ratio) and C26:0-LPC analysis in plasma and DBS, we measured these metabolites in samples from controls (*n* = 67), ALD males (*n* = 26), ALD females (*n* = 19) and ZSD patients (*n* = 35) (Figure 2). For C26:0, the median level in controls was 0.67 μmol/L (range 0.37–1.34 μmol/L), in ALD males it was 2.92 μmol/L (range: 1.19–5.01 μmol/L), in ALD females it was 1.81 μmol/L (range 1.11–4.06 μmol/L) and in ZSD patients it was 2.41 μmol/L (0.95–9.74 μmol/L). The upper limit of the reference range in our laboratory for C26:0 in plasma is 1.32 μmol/L. One out of 67 controls had C26:0 levels above the reference range. One out of 26 ALD males, 1/19 ALD females and 3/35 ZSD patients had a C26:0 concentration in the reference range. For the C26:0/C22:0 ratio, the median ratio in controls was 0.012 (range 0.008–0.053), in ALD males it was 0.055 (range: 0.033–0.09), in ALD females it was 0.03 (0.02–0.05) and in ZSD patients it was 0.05 (range: 0.02–0.39). The upper limit of the reference range in our laboratory for the C26:0/C22:0 ratio in plasma is 0.02. Six out of 67 controls had a C26:0/C22:0 ratio above the upper limit of the reference range. The elevated C26:0/C22:0 ratio of these samples was the result of relatively low C22 concentration [range: 12–59 μmol/L (reference range: 40–119 μmol/L)] in combination with normal C26 concentration (range: 0.61–1.29 μmol/L). A peroxisomal disorder diagnosis was rejected in these six control individuals because all other peroxisomal parameters (phytanic acid, bile acid intermediates, and plasmalogens) were normal, and VLCFA analysis in a repeat sample showed no abnormalities. None of the ALD males, 1/19 ALD females and 2/35 ZSD patients had a C26:0/C22:0 ratio within the reference range. For C26:0-LPC measurements in DBS and plasma, the median level in controls was 0.037 μmol/L (range: 0.011–0.063 μmol/L), in ALD males it was 0.467 μmol/L (range: 0.190–1.004 μmol/L), in ALD females it was 0.266 μmol/L (range: 0.118–0.576 μmol/L) and in ZSD patients it was 0.453 μmol/L (range: 0.074–2.485 μmol/L). The upper limit of the reference range in our laboratory is 0.072 μmol/L. All controls had a C26:0-LPC concentration below this upper limit and all patients had C26:0-LPC levels above the upper limit of the reference range. In this cohort, only C26:0-LPC showed a complete separation between controls and patients.

**FIGURE 3:**
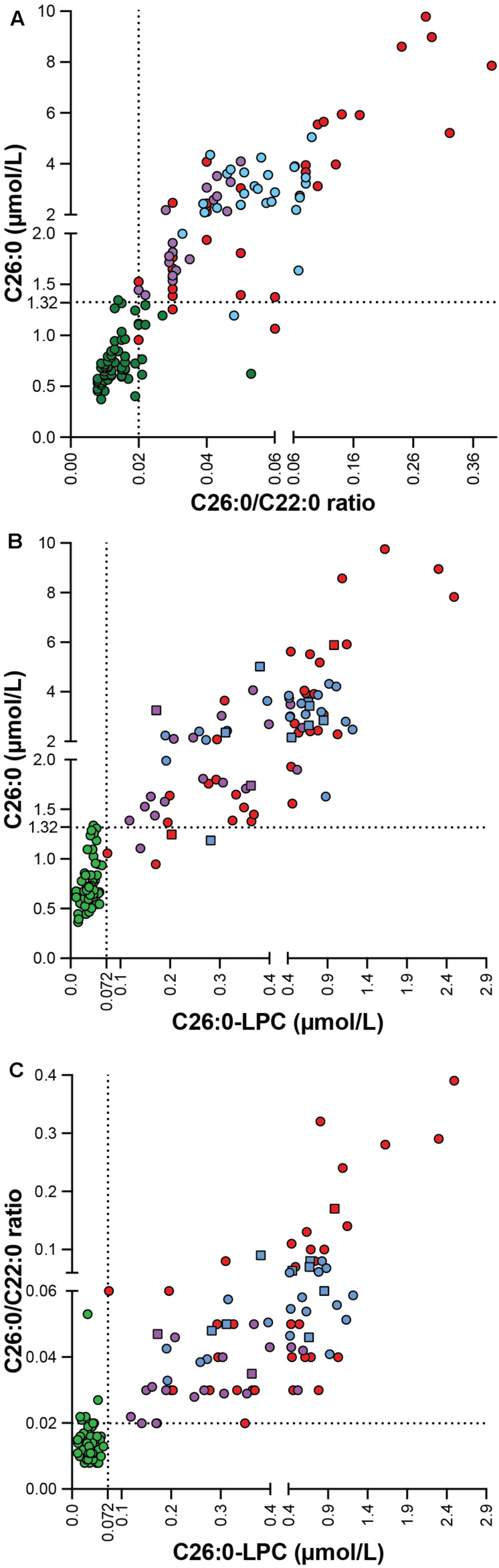
Correlation plot showing the correlation between C26:0 concentration and the C26:0/C22:0 ratio **(A)**, C26:0-LPC and C26:0 levels **(B)** and C26:0-LPC level and the C26:0/C22:0 ratio **(C)** in controls (green, *n* = 67), ALD males (blue, *n* = 26), ALD females (purple, *n* = 19) and ZSD patients (red, *n* = 35). C26:0-lysoPC results from DBS are indicated by squares and plasma by circles. The upper limit of the reference range for C26:0 (1.32 μmol/L), the C26:0/C22:0 ratio (0.02) and C26:0-LPC (0.072 μmol/L) is indicated by the dashed lines.

Since the C26:0-LPC and VLCFA analyses were performed on samples generated from the same blood sample, we investigated the correlation between the 3 diagnostic markers with a Spearman’s rank-order correlation (Figure 3). When, combining controls and patients there was a strong positive correlation between all 3 biomarkers. For the C26:0/C22:0 ratio and C26:0 concentration the Spearman’s rank correlation coefficient was *r* (147) = 0.892, *p* < 0.001; for C26:0-LPC and C26:0 levels it was *r* (147) = 0.892, *p* < 0.001; and for C26:0-LPC and the C26:0/C22:0 ratio it was *r* (147) = 0.816, *p* < 0.001.

## Discussion

### Comparison of C26:0-LPC Levels in DBS and Plasma

The first objective of this study was to investigate the correlation between C26:0-LPC levels measured in DBS and plasma. To this end, we compared C26:0-LPC in DBS and plasma samples that were derived from the same blood sample. Our results demonstrate that both in control and patient samples C26:0-LPC levels in DBS and in plasma are very strongly correlated (*r* (114) = 0.962, *p* < 0.001). C26:0-LPC in DBS is an effective biomarker for ALD and ZSD patients (Hubbard et al., 2006, 2009; Huffnagel et al., 2017; Klouwer et al., 2017). In some cases, DBS sampling provides considerable advantages over plasma. DBS sampling is relatively non-invasive compared to whole blood sampling. It can be performed in a patient’s home or at a local point of care, and requires less personnel training (McDade et al., 2007). DBS can be easily shipped by regular mail which is much cheaper and less logistically challenging than transporting plasma or whole blood, which requires specific supply chain conditions by courier, such as dry ice for plasma or controlled temperature for whole blood. Despite these advantages DBS do have diagnostic limitations compared to plasma. For a complete diagnostic testing for peroxisomal disorders not only VLCFAs analysis is required, but also phytanic acid, pristanic acid, di- and trihydroxycholestanoic acid analysis in plasma and plasmalogen analysis in erythrocytes. Because currently there is no evidence that these tests can be done in DBS, plasma is still needed in the diagnostic work-up for patients with peroxisomal disorders.

### Comparison of C26:0-LPC and VLCFA

Traditionally, VLCFA analysis plays an important role in the diagnosis of peroxisomal disorders. However, the analysis of VLCFAs using GCMS is time-consuming and labor intensive. C26:0-LPC is a potential alternative marker for VLCFAs. Therefore, the second objective of this study was to compare the diagnostic performance of VLCFA and C26:0-LPC analysis and systematically evaluate potential differences in outcome between these markers. To this end, we measured VLCFA levels and C26:0-LPC in samples from controls, ALD patients (males and females) and ZSD patients. Almost all patient samples showed increased C26:0/C22:0 ratios. None of the ALD males, 1/19 ALD females and 2/35 ZSD patients had a C26:0/C22:0 ratio within the reference range. The finding that 6 samples from the control group fell outside the reference range of C26:0/C22:0 indicates that false positives should be considered. For these samples the false positive results were due to low C22:0 and normal C26:0 concentrations. For this reason, plasma VLCFA analysis always includes both the analysis of C26:0 and the C26:0/C22:0 ratio as a means of improving the accuracy of the readout. The combination of these 2 markers increases the sensitivity of the analysis. Furthermore, false-positive results in VLCFA levels have been reported in individuals without a peroxisomal defect. For example, diabetic ketoacidosis, a ketogenic diet or hemolysis of the blood sample can result in false-positive VLCFA levels (Kishimoto et al., 1980; Brown et al., 1982; Theda et al., 1993). Importantly, non-fasted blood samples obtained from individuals who had consumed peanut butter in the hours prior to blood collection have VLCFA levels that are in the abnormal range (Lam et al., 2012). This is most likely due to direct dietary effects on plasma components that contain VLCFA, including lipoprotein particles and cholesterol esters (Engelen et al., 2010; Wanders et al., 2010). This dietary influence on C26:0 levels is minimized in practice by blood sampling in a fasted state. However, fasting is a considerable hindrance for the patient and non-compliance might occur. There is currently no evidence that C26:0-LPC levels are affected by diet. In blood, C26:0-LPC is primarily found as a membrane component in cells such as erythrocytes (Tanaka et al., 1989). Since erythrocytes cycle for 2 to 4 months, the C26:0-LPC level is expected to be relatively stable over this period with little effect of dietary influences (Cohen et al., 2008; Mock et al., 2011). This simplifies sampling procedures and results in a lower number of false positives. Indeed, all C26:0-LPC levels measured in control samples in this study were within reference range. All ALD and ZSD samples had increased C26:0-LPC levels. Overall our data show that C26:0-LPC measurement has a superior diagnostic performance compared to the traditional VLCFA analysis in all patient groups and results in less false positives. Consistent with earlier work, males tended to have higher C26:0 and C26:0/C22:0 levels than females (O’Neill et al., 1984; Moser et al., 1999; Engelen et al., 2014), which was also observed for C26:0-LPC. It has been well-established that plasma VLCFA analysis may result in a false negative result in approximately 15–20% of women with ALD (Moser et al., 1999). The results of this study are in agreement with our earlier work showing that women with ALD have elevated C26:0-LPC, including those with normal plasma C26:0 and C26:0/C22:0 (Huffnagel et al., 2017, 2019). Taken together these results show that the analysis of C26:0-LPC, either in DBS or in plasma, considerably increases the sensitivity of detecting disorders associated with a defect in peroxisomal beta oxidation. Albeit, some caution is warranted with the interpretation of a normal C26:0-LPC result given the fact that the lowest level measured in a DBS sample from an ALD female (0.080 μmol/L) and a plasma sample from a ZSD patient (0.074 μmol/L) were very close to the upper limit of the reference range (0.072 μmol/L) (Figure 1). In line with plasma VLCFA levels, C26:0-LPC levels in ALD males and females overlap with the levels measured in ZSD patients. No differentiation can be made between ALD patients and ZSD patients based on either C26:0, C26:0/C22:0 or C26:0-LPC level alone. Analysis of other metabolites and genetic analysis are required to differentiate between these disorders and confirm the diagnosis. Besides a superior diagnostic performance, the LCMS-based analytical method for C26:0-LPC is far less time consuming and labor intensive than the GCMS-based VLCFA analysis. Increased C26:0 levels have been reported in peripheral blood from patients with Alzheimer’s disease and vascular dementia (Lizard et al., 2012; Zarrouk et al., 2015). It would be interesting to evaluate C26:0-LPC in these patients.

In conclusion, this study demonstrates that C26:0-LPC analysis performed in DBS and in plasma are strongly correlated. Furthermore, our results demonstrate the analysis of C26:0-LPC has superior diagnostic performance for ALD and ZSD patients compared to C26:0 and C26:/C22:0. Based on our results we recommend implementation of C26:0-LPC analysis in DBS and/or plasma in the diagnostic work-up for peroxisomal disorders.

## Data Availability Statement

The raw data supporting the conclusions of this article will be made available by the authors, without undue reservation.

## Author Contributions

YJ, SF, WK, ME, SG, FV, and SK conceived the project. ID and HL performed the VLCFA and C26:0-LPC analyses. YJ and SK performed the data analyses. YJ, SF, RB, ME, SG, FV, and SK wrote the manuscript. All authors contributed to the article and approved the submitted version.

## Conflict of Interest

The authors declare that the research was conducted in the absence of any commercial or financial relationships that could be construed as a potential conflict of interest.
